# *Does Croton Argyrophyllus* Extract Has an Effect on Muscle Damage and Lipid Peroxidation in Rats Submitted to High Intensity Strength Exercise?

**DOI:** 10.3390/ijerph16214237

**Published:** 2019-10-31

**Authors:** Silvan Silva de Araújo, Felipe José Aidar, Dihogo Gama de Matos, Jymmys Lopes dos Santos, Lúcio Marques Vieira Souza, Albená Nunes da Silva, Rodrigo Miguel dos Santos, Anderson Carlos Marçal, Daniella Mota Mourão, Amário Lessa Júnior, Geraldo Magela Durães, André Luiz Gomes Carneiro, Rodrigo Gonçalves da Silva, Mauro Martins Teixeira, Charles dos Santos Estevam

**Affiliations:** 1 Post-Graduate Program in Physical Education, Federal University of Sergipe, São Cristóvão SE 49100-000, Brazil; silvan.ssa@gmail.com (S.S.d.A.); acmarcal@yahoo.com.br (A.C.M.); 2 Post-Graduate Program in Physiological Sciences, Federal University of Sergipe, São Cristóvão SE 49100-000, Brazil; rms.edf@hotmail.com; 3 Group of Studies and Research of Performance, Sport, Health and Paralympic Sports—GEPEPS, Federal University of Sergipe, São Cristovão, Sergipe 49100-000, Brazil; dihogogmc@hotmail.com; 4 Department of Physical Education, Federal University of Sergipe, São Cristóvão, Sergipe 49100-000, Brazil; 5 Department of Physiology, Federal University of Sergipe, São Cristóvão, SE 49100-000, Brazil; cse.ufs@gmail.com; 6 Institute of Parasitology, McGill University, Montreal, QC H9X 3V9, Canada; 7 Post-Graduate Program in Biotechnology, Northeast Network in Biotechnology (RENORBIO), Federal University of Sergipe, São Cristóvão SE 49100-000, Brazil; jymmyslopes@yahoo.com.br (J.L.d.S.); profedf.luciomarkes@gmail.com (L.M.V.S.); 8 Exercise’s Inflammation and Immunology Laboratory, Sports Center, Federal University of Ouro Preto, Minas Gerais 35400-000, Brazil; albenanunes@hotmail.com; 9 Department of Circulation and Medical Imaging, St. Olav’s Hospital, Norwegian University of Science and Technology (NTNU), NO-0508 Trondheim, Norway; 10 Department of Medical Clinic, State University of Montes Claros, Montes Claros, MG 39401-089, Brazil; daniella_mourao@hotmail.com; 11 Department of Physical Education, State University of Montes Claros, Montes Claros, MG 39401-089, Brazil; lessauni@gmail.com (A.L.J.); gmdmoc@yahoo.com.br (G.M.D.); algcarneiro@gmail.com (A.L.G.C.); 12 Department of Physical Education, University Funorte of Montes Claros, Montes Claros, MG 39401-089, Brazil; rodrigo.edfisio@yahoo.com.br; 13 Department of Pathology, Institute of Biological Sciences, Federal University of Minas Gerais, Belo Horizonte 31270-901, Brazil; mmtex.ufmg@gmail.com

**Keywords:** strength exercise, croton, oxidative stress, muscle damage, lipoperoxidation, hidroethanol extract

## Abstract

Many species of the genus *Croton* have been used for anti-inflammatory, antiproliferative, antidiabetic, and antitumor purposes. The objective was to evaluate the effect of a hydroethanolic extract (HEE) from the inner bark of *Croton argyrophyllus* (Euphorbiaceae) on muscle damage and oxidative stress in rats after high intensity exercise. The animals were divided into four groups: (i) the sedentary group (SV; *n* = 7), (ii) the exercise vehicle group (EV, *n* = 7), (iii) the sedentary group HEE (SHG; *n* = 7) composed of sedentary animals and treated with the hydroethanolic extract of *C. argyrophyllus* (200 mg/kg, v.o.), and (iv) the HEE exercise group (HEE; *n* = 7) composed of animals submitted to resistance exercise (RE) and treated with the hydroethanolic extract of *C. argyrophyllus* (200 mg/kg, v.o.). In the 2,2-Diphenyl-1-picrylhydrazyl (DPPH) test, the HEE showed lower values of inhibition potential (IP%) at 39.79% compared to gallic acid, 87.61%, and lipoperoxidation inhibition at 27.4% (100 µg/mL) or 28.6% (200 µg/mL) (*p* < 0.001). There was inhibition in free radicals in vivo. The HEE of *C. argyrophyllus* partially reduced the biomarkers of oxidative stress in muscle tissue and muscular damage (creatine kinase (CK) and Lactate Dehydrogenase (LDH)) (*p* < 0.05) in rats, and in this sense it can be an aid to the recovery process after exhaustive efforts.

## 1. Introduction

Resistance exercise (RE) is characterized as a physical activity involving voluntary actions of the skeletal muscle in a given body segment against an external resistance [1]. The execution of high intensity or exhaustive RE can result in injuries and chronic fatigue in part due to the imbalance between the production of reactive oxygen species (ROS) and endogenous antioxidant activity [2]. Although it is established that optimal production of ROS is important to induce muscle contraction, high concentrations of ROS accelerate the process of exercise-induced muscle fatigue [3,4].

Some environmental factors can challenge the cellular antioxidant defense system, including physical exercise, and especially high intensity exercise. Accordingly, exhaustive exercise sessions lead to an imbalance between the antioxidant system and the free radicals formed during the oxidative metabolism due to ischemia-reperfusion during each set of cyclical contractions [5]. In turn, oxidative stress can cause damage to cell structures and macromolecules such as lipids, proteins, and nucleic acids, which result in a loss of physical performance and muscle injuries [2,5,6].

The physiological functionality of the systems during intense exposure to acute exercise can be extended by supplemental antioxidants, such as those found in medicinal plants [7]. Since free radicals are unstable molecules that feature unpaired electrons, they can be inhibited by isolated plant phenolic compounds, such as flavonoids [7]. These substances, due to their redox ability, can neutralize oxidative free radical activity [8,9], reinforcing the cellular antioxidant system composed of the enzymes superoxide dismutase (SOD), catalase (CAT), and the system glutathione peroxidase/glutathione reductase (GPX/GR), which maintains an appropriate balance between catabolic and anabolic processes [10].

*Croton argyrophyllus* extracts are widely distributed and consumed therapeutically in the semi-arid northeast. In addition, two new casbane diterpenes (1 and 2) of *Croton argyrophyllus*, an abundant shrub in the northeastern region of Brazil, have been reported to have been isolated, which has shown a marked increase as an ethnobotanical treatment of heart diseases and as a tranquilizer [11], although there is still much to clarify in regards to the protective redox effects of their extracts and other secondary metabolites on oxidative stress related to exercise. In this context, the objective of this study was to evaluate the effect of a crude extract from the inner bark of *C. argyrophyllus* on on muscle damage and oxidative stress in female rats performing high-intensity exercises.

## 2. Materials and Methods

### 2.1. Plant Material Collection

The inner bark of the plant *C. argyrophyllus* (1.5 kg) was obtained in the city of Olho d’Água do Casado, Alagoas (09°25’00” S, 38°01’11” W). It was then subjected to oven drying (37 °C), crushed, and reduced to a powder. The resulting powder was macerated in ethanol (90% v/v) at 25 °C yielding the hydroethanolic extract (HEE). The HEE was concentrated in a rotary evaporator (Logen Scientific^®^, Lagos, Nigeria) under reduced pressure at 50 °C until achieving the final desired volume, yielding 73.3 g of HEE. The dried extract was stored in a glass bottle and preserved in a freezer at −30 °C.

### 2.2. High-Performance Liquid Chromatography (HPLC)

To test for the presence of organic compounds available in the HEE, we used a Shimadzu high performance liquid chromatography (HPLC) (Prominence model, Kyoto, Japan). This HPLC consists of a model DGU-20A3 vacuum cleaner, LC-6A high pressure pumps, and photodiode array (PDA) detection system coupled with Interface CBM 20A. Data acquisition and processing was performed using LC Solution software. The analysis was performed on a Phenomenex LUNAs analytical column C18 column (250 × 4.6 mm i.d., 5 μm particle diameter, Phenomenex, Torrance, CA, USA). Separations of the components were performed on a reverse phase elution gradient, which emphasizes the polar compounds [12]. To that end, 20 μL of HEE (1 mg·mL^−1^), previously membrane filtered at 0.20 μm in diameter, was injected into the mobile phase HPLC, which consisted of water and acetonitrile, both acidified with acetic acid. The elution was characterized as isocratic: 70:30:0.5%. The detection was performed in the UV spectra and obtained at a range of 250 nm to 350 nm.

### 2.3. Antioxidant Activity In Vitro

HEE was dissolved in methanol to obtain a stock solution of 500 µg/mL, from which aliquots were removed and added to a solution of 2,2-diphenyl-1-picrilhidrazina (DPPH) (40 µg/mL) at concentrations of 60, 90, 120, 150, and 180 µg/mL. The dilutions of the positive control, gallic acid, led to concentrations of 1–10 µg/m, with all reaction solutions in a final volume of 3 mL. A mixture of methanol (2.7 mL) and HEE (0.3 mL) was used as the blank. Measures of absorbance were made at 515 nm at 1, 5, and 10 min, and then every 10 min up to 1 h [13]. The percentage of the remaining DPPH (DPPHREM%) was calculated according to the method of Souza et al., [13] using the equation: %DPPHREM = (DPPH)T/(DPPH)T0 × 100, where (DPPH)T is the concentration of DPPH in the middle, after the reaction with the sample, and (DPPH)T0 is the initial concentration of DPPH, namely 40 µg/mL (100 mmol/mL). The effective concentration required to decrease the initial concentration of DPPH by 50% (EC50) was calculated by plotting the %DPPHREM over 60 min in opposition to the sample concentrations. The results are expressed in µg/mL ± standard deviation. The higher the consumption of DPPH per sample, the lower its EC50, and the higher its antioxidant activity. The ratio of the DPPH concentration to the EC50 can be obtained by the antioxidant activity index (AAI = (DPPH)/EC50), which Scherer and Godoy [14] consider the best expression of the antioxidant capacity.

### 2.4. Lipoperoxidation

The ability to inhibit lipid peroxidation was determined by measurement of thiobarbituric acid reactive substances (TBARS) in a test tube, and a lipid matrix composed of egg yolk (1 mL) was homogenized in 99 mL of phosphate buffer 7.4 (20 mM) [15]. The quantification of TBARS was performed according to Lapenna et al. [16] and Sanocka and Kurpisz [17]. The test tubes containing the reaction mixtures were incubated at 37 °C for 30 min to generate oxidative damage. Then, 500 µL of trichloroacetic acid (TCA 15%) was added for precipitation of the proteins, which were removed by centrifugation (2000 rpm, 10 min). The supernatant was collected and 500 µL of thiobarbituric acid (TBA 0.67%) was added to the test tubes. These were incubated at 95°C for 60 min. Then, absorbance measurements were performed at a wavelength of 532 nm.

All tests were performed in triplicate and compared to the standard antioxidant performance, trolox, at the same concentrations of the samples. The molar extinction coefficient 1.54 × 105 M^−1^ cm^−1^ and TBARS was expressed as nmol Eq. MDA mL^−1^ plasma.

### 2.5. Experimental Group

**Figure 1:**
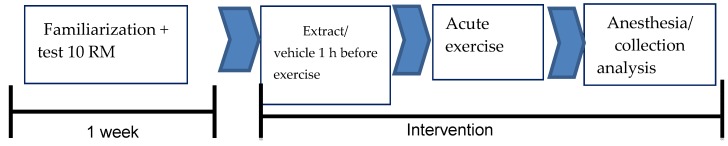
Diagram of the experimental protocol.

The animals were divided into four groups: (i) the sedentary vehicle group (SG; *n* = 7) composed of sedentary vehicle-treated animals (Tween 80, orally (v.o.)), (ii) the exercise vehicle group (EG, *n* = 7) composed of animals treated with the vehicle (Tween 80, v.o.) and submitted to RE, (iii) the sedentary group HEE (SHG; *n* = 7) composed of sedentary animals and treated with the hydroethanolic extract of *C. argyrophyllus* (200 mg/kg, v.o.), and (iv) the HEE exercise group (EHG; *n* = 7) composed of animals submitted to RE and treated with the hydroethanolic extract of *C. argyrophyllus* (200 mg/kg, v.o.) see Figure 1.

### 2.6. Resistance Exercise Model Adapted to Rats

The hind limbs of rats in the exercise groups were submitted to a single intense workout in a weight lifting technique previously described in detail [18]. Each animal was wearing a leather jacket attached to a wooden bar (support), which was connected at the other end to a fulcrum fixed to a wooden base. The animal, therefore, from an upright and supported position with its back foot on the table, ran to full extension of its legs (looking for a jump), and then was electrically stimulated in its tail (10 V, 100 Hz, for 3 s). For electrical stimulation, we used self-adhesive electrodes (ValuTrode brand, model CF3200, Santa Tereza do Oeste, Brazil, size 3.2 cm, placed on the tail and connected to a stimulator) (BIOSET, Physiotonus Four, Model 3050, Rio Claro, São Paulo, Brazil). These parameters were adopted because they are associated with changes in the levels of catecholamines, sympathetic activity, and adrenal hypertrophy [19].

The overhead consisted of lead plates embedded in the support. The animals were previously adapted to the device for a week, including those groups not exercised (VS and ES). Supplementation with hydroethanolic extract of *C. argyrophyllus* was began one hour before the start of the session. The session involved multiple sets of 10 reps with a 1 min interval between each series until exhaustion. The first set surveys were given with a load of 500 g. In subsequent sets, a load of 500 g was added until the rat could not complete 10 repetitions. In this case, the load was then adjusted in increments or decreases of 100 g to meet the maximum load when 10 repetitions could be performed, and then the maximum load of 10 repetitions (10 RM) was determined. The 10 RM was repeated until the animal could not complete one set of 10 repetitions, and then the load was reduced by 500 g. This procedure was maintained until the animal could not complete three consecutive sets, even though the charge was reduced.

### 2.7. Sacrifice and Collection of Biological Material

Collection and preparation procedures of biological material for analysis of tissue injury markers and evaluation of some of the determinant markers of oxidative stress were performed according to the methodology described by Halliwell and Gutteridge [20].

At the end of the workout, the animals were anesthetized with sodium thiopental (40 mg/kg, ip) for blood collection by cardiac puncture, and then were euthanized and decapitated. Blood was immediately centrifuged at 800 g for 15 min at 4 °C, and the supernatant was stored at −80 °C for further analysis.

### 2.8. Determination of TBARS

An aliquot of a 20 µL plasma was added to 40 µL of a mixture consisting of equal parts of trichloroacetic acid (TCA), 15% 0.25 N HCl, 0.375% TBA, 2.5 mM butylated hydroxytoluene (BHT), and 40 µL of sodium dodecyl (SDS) at 8.1%, and then it was heated at 95 °C for 30 min. The pH of the mixture was 0.9. BHT was used to prevent lipid peroxidation during heating. After cooling to room temperature, 40 µL of n-butanol was added, and then the mixture was centrifuged at 800 g for 15 min for further evaluation by spectrophotometry [16]. As a background control, a system with 40 µL formed of equal parts of 15% TCA, 0.25 N HCl, 2.5 mM BHT, and 40 µL of 8.1% SDS was used. The spectrophotometric reading of the samples was taken at 532 nm, representing the peak complex formed between the absorption and dialdehyde acid (TBA-MDA). A molar extinction coefficient of 150,000 M^−1^cm^−1^ was used [16].

### 2.9. Quantification of Plasma and Muscle Total Creatine Kinase

Acute resistance exercise involving maximum repetitions until volitional exhaustion can induce micro injuries in muscle fibers [21]. Accordingly, the activity of creatine kinase (CK) [22], the regulatory enzyme controlling the resynthesis of adenosine triphosphate (ATP), can be used as an indicator of muscle damage after an acute exercise session [22], as adopted in the present study.

For quantification, the recommendations of the commercial kit manufacturer were used (Labtest^®^; Lagoa Santa—Minas Gerais, Brazil), where 20 µL plasma from each animal was homogenized in specific reagents at 37 ± 0.2 °C and then read in a spectrophotometer UV/VIS at 340 nm.

### 2.10. Quantification of Lactate Dehydrogenase Total Plasma

For the quantification of LDH, the recommendations were used as provided by the commercial kit manufacturer (Labtest; Lagoa Santa-MG). Like CK, the levels of lactate dehydrogenase (LDH) are also susceptible to high intensity exercise stimuli, and they can be used to indicate the plasma dosage status for the intactness of the muscle membrane.

LDH is present in nearly all organs and tissues of the organism and its catalytic activity is due to the presence of several isoenzymes, which can form different patterns depending on the source of LDH present in the serum. In the conditions prescribed by the kit’s manufacturer, LDH catalyzes the conversion of pyruvate to lactate as NADH is oxidized to NAD^+^. The catalytic activity is determined by the speed of NADH’s disappearance. This activity was quantified according to the manufacturer’s recommendations as contained in the commercial kit (Labtest^®^), in which 20 µL of plasma of each animal was homogenized in specific reagents at 37 ± 0.2 °C and read on a spectrophotometer UV/VIS at 340 nm.

### 2.11. Statistical Analysis

The results were presented as mean ± standard error of the mean (SEM). They are considered to be statistically significant results with a probability of error less than 5% (*p* < 0.05). Normality, homoscedasticity, and variance tests were performed simultaneously using Prism software, version 5.0 (GraphPad Software, San Diego, CA, USA). The analysis of variance (ANOVA) was used followed by a multiple comparison test, as appropriate. The EC50 (effective concentration to neutralize DPPH) was used in the in vitro tests.

## 3. Results

**Figure 2:**
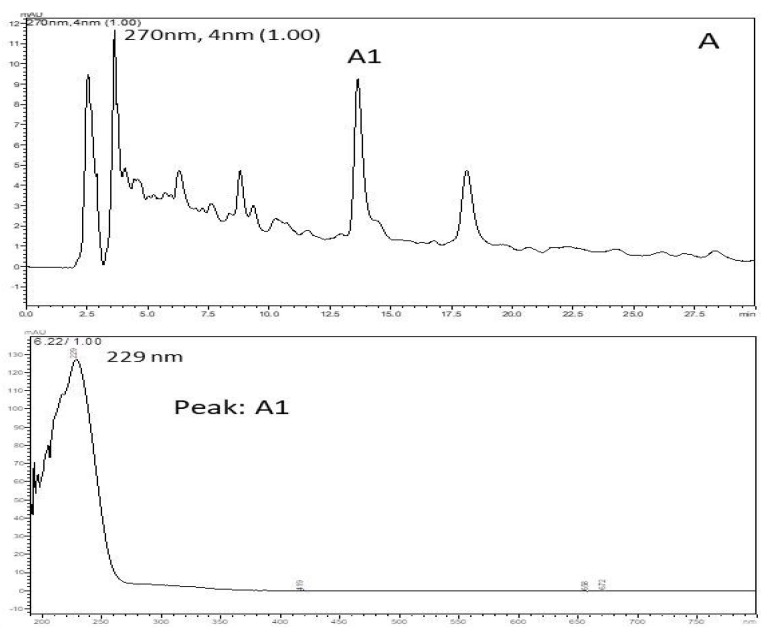
Chromatographic profile HPLC/PAD of the hydroethanolic extract of *C. argyrophyllus*. It shows the spectrum UV/VIS of the prominent peak (absorption band at 229 nm) and retention time.

Based on retention time and PAD spectrum, the HEE showed an identical chromatographic behavior to the polar compounds. In this sample (Figure 2), retention time (RT) was recorded for 13 min and the greatest peak intensity showed a higher UV absorption at 229 nm (Peak A1) and a run time of 30 min. The chromatographic analysis serves to show the complexity of the compound, which allows us to evaluate the purity of the sample; it was recorded from 250 to 350 nm using LC-PAD, and gives us an indication of the presence of the phenolic compounds.

The x-axis is time and the y-axis is intensity. Thus, the figure shows that retention was recorded at 13 min. On the other hand, there was UV absorption intensity at 229 nm. This variation occurred during the total time of 30 min. Therefore, during 30 min of execution, the absorption peak occurred at 13 min with an intensity of 229 nm, with no other important values during the execution time.

The polarity, as phenolic compounds present in plant extracts, is directly related to their antioxidant potential due to the amount of hydroxyl groups [12]. The inhibitory potential (IP) was lower than the positive control gallic acid (Table 1). This parameter was confirmed with a higher EC_50_ value, which is the required concentration of the test compound to reduce by 50% the initial concentration of DPPH in regards to the content of the antioxidant activity index (AAI), which qualifies the samples by their strength to help neutralize the DPPH radical, according to Scherer et al. [14].

Values with different symbols indicate differences between the samples and gallic acid (*p* < 0.05): HEE: hydroethanolic extract, IP%: inhibitory potential, and EC_50_: AAI: antioxidant activity index. The IP% and EC_50_ of the sample were calculated at their respective time of 30 min. One-way ANOVA was followed by a Tukey test (ρ < 0.05). The AAI classifies the sample as weak when AAI < 0.5, moderate when 0.5 < AAI < 1, strong when 1 > AAI < 2, and very strong when IAA > 2.

**Figure 3:**
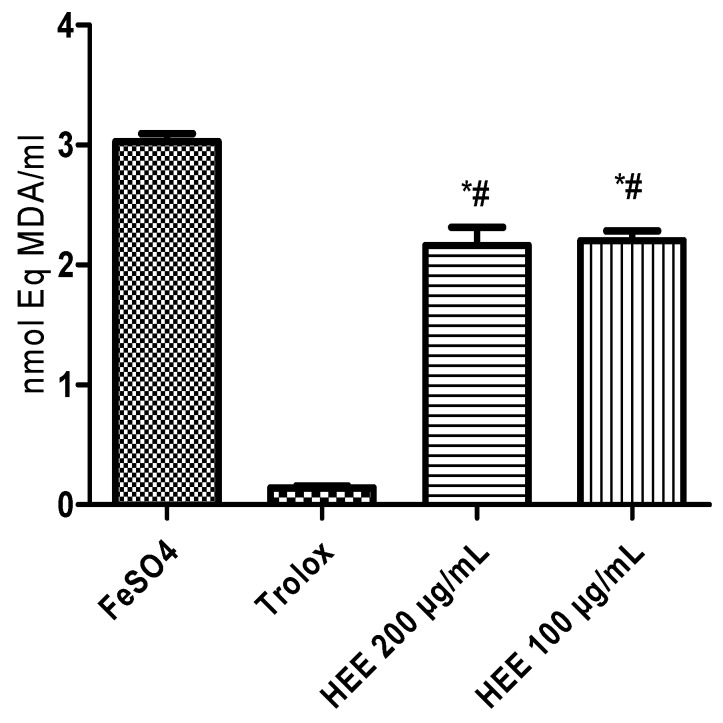
Inhibitory capacity of lipid peroxidation measured by the generation of malondialdehyde (MDA) induced by ferrous sulfate. * Different from the negative control (*p* < 0.001); # Different from the positive control trolox (*p* < 0.0001).

The decrease in lipid oxidation by free radicals induced by ferrous sulfate was evaluated by the reduction in the level of TBARS formation. The different concentrations applied reduced the TBARS levels compared to the negative control (Figure 3). This performance represents a lipoperoxidation inhibition of 27.4% (100 µg/mL) and 28.6% (200 µg/mL).

**Figure 4:**
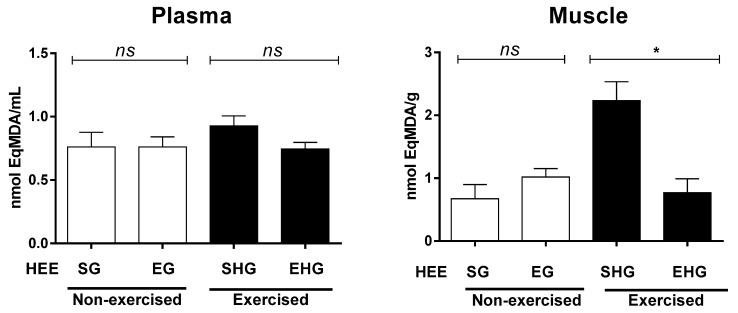
Concentration of malondialdehyde in animals subjected to the administration of hydroethanolic extract of *C. argyrophyllus* (EHE) and further execution of acute resistance exercise. ns: not significant; * differences between the exercised groups. The results are shown as mean ± SEM. One-way ANOVA with a Bonferroni post test; *p* < 0.05. SG—sedentary vehicle group; EG—exercise vehicle group; SHG—sedentary group HEE; EHG—HEE exercise group.

The plasma lipid peroxidation was assessed by quantification of the TBARS, which is represented here by malondialdehyde (MDA). There were no exercise group differences in plasma MDA (Figure 4). However, when the MDA levels were measured in the gastrocnemius muscle (Figure 4), it was found there were significant differences only in the exercised group, with reduced MDA levels in animals treated with HEE, suggesting that the HEE reduced the oxidative stress induced by RE until exhaustion.

**Figure 5:**
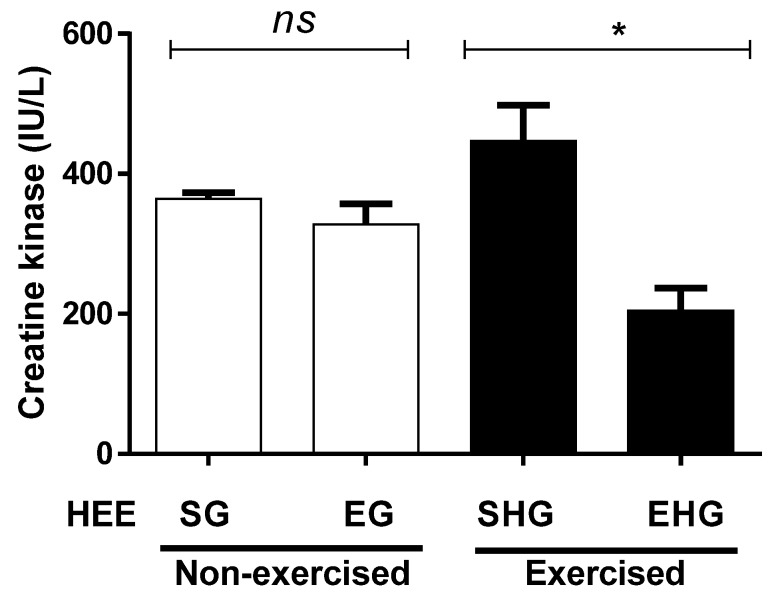
Plasma concentration of creatine kinase in animals exercised and subjected to the administration of hydroethanolic extract of *C. argyrophyllus* (HEE). The results are presented as mean ± SEM and evaluated by one-way ANOVA with a Bonferroni post test; *p* < 0.05. ns: not significant; * differences between the exercised groups. SG—sedentary vehicle group; EG—exercise vehicle group; SHG—sedentary group HEE; EHG—HEE exercise group.

Figure 5 illustrates the quantification of plasma CK. No significant differences were observed between the non-exercised groups, treated (HEE), or vehicle groups (Tween 80). However, the plasma concentration of CK in the exercised group treated with EHE decreased (54.13%) compared with the exercised group treated with the vehicle only.

**Figure 6:**
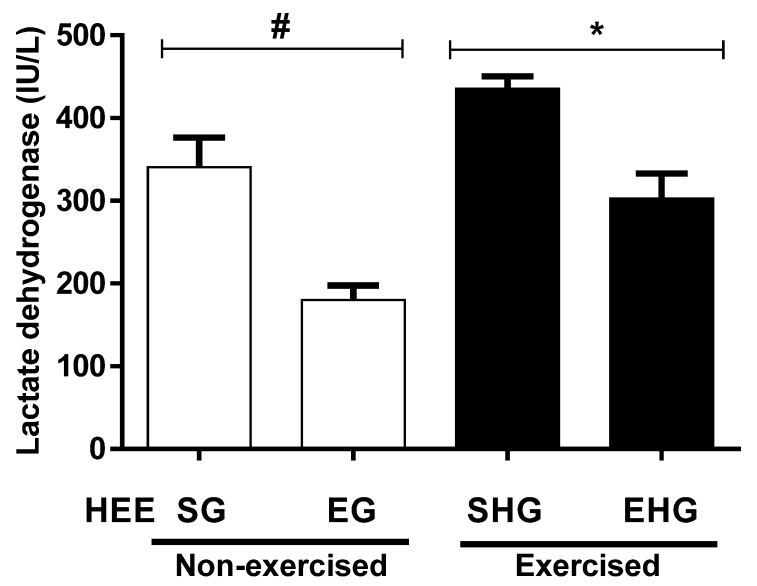
Evaluation of lactate dehydrogenase (LDH) plasma in animals subjected to the administration of hydroethanolic extract of *C. argyrophyllus* (HEE) and further execution of acute resistance exercise. The results are presented as mean ± SEM and evaluated by one-way ANOVA with a Bonferroni post test; *p* < 0.05. # SG—sedentary vehicle group; EG—exercise vehicle group; SHG—sedentary group HEE; EHG—HEE exercise group.

The plasma concentrations of LDH are shown in Figure 6. It was found that there was a reduction in the plasma concentration of LDH (47.14%) in the non-exercised groups treated with EHE compared to vehicle. In addition, the plasma concentration of LDH in the exercised group and the one supplemented with EHE also showed a reduction (30.53%) compared with the vehicle group exercised.

## 4. Discussion

Hydroethanolic extract of *C. argyrophyllus* has antioxidant effects and is able to reduce the damage caused by acute sessions of RE until exhaustion. Possibly, this ability can be attributed to the compounds of a phenolic nature present in its composition [23,24], as shown in the chromatographic analysis by HPLC. The high affinity and hydrophilicity of the compounds present in plant extracts and fractions are directly related to their antioxidant capacity due to the diverse nature of hydroxyl groups [25].

The antioxidant ability of the extract from *C. argyrophyllus* to neutralize the synthetic free radical DPPH was tested. Although the EC50 extract had a higher activity than that of the gallic acid positive control, it was comparable with *Croton* extracts from other species, as seen in the study by Ndhala et al. [26], which showed that *C. zambesicus’s* EC50 was 1018 µg/mL. Regarding the IP%, HEE from *C. argyrophyllus* met the same standards found by Furlan et al. [24] in nine species of *Croton* from Argentina.

This effect of the extract of *C. argyrophyllus* is important because lipid peroxidation is involved in the ROS reaction with the polyunsaturated fatty acid constituents of cell membranes, which can change the lipid structures, proteins, carbohydrates, and DNA, as well as present carcinogenic, mutagenic, and protein denaturant properties [27,28].

In previous studies [7,26], the ability of *Croton* extracts to resist against lipid peroxidation has been tested, which was corroborated in the present study where the generation of malondialdehyde was significantly inhibited. With the confirmation of the antioxidant capacity of *C. argyrophyllus* extract, it was possible to assess the extract’s ability to reduce the damage caused by a session of resistance training. Exercise, when done exhaustively or at intensities above the body’s organic capabilities, can be an inducer of ROS production [10]. In a study by Coombes et al. [29], due to the supplementation with antioxidants, they found low levels of oxidative stress, although the production of contractile force was reduced. This evidence can be explained by the reduced generation of MDA, which is a product that can damage the lipid membranes of the muscles. However, in that present study, there was no evaluation of the absolute levels of force in the exercised groups.

Several substances in the extract, such as phenols and terpenoids, synergistically may present redox activity in their electron donor groups [25], although this effect has not been observed at plasma levels. Thus, there are possibilities to use other dosages in future studies, beyond those used here, in addition to chronic supplementation.

The adaptation to exercise favors greater stabilization of the cell membrane when subjected to stress. This is due, among other factors, to the homeostasis in the synthesis and resynthesis of ATP for reducing the oxidative damage [30], which can be covered by supplementing with antioxidants and nutrients that can be turned into energy-rich substrates.

As seen in this study, administration of HEE to rats that performed high-intensity RE provided a lower plasma concentration of CK, which can be justified by the mechanism related to cellular energy homeostasis [2] when considering the case of acute exercise, which does not provide for adaptation to physical stress. Although the CK levels were above the reference values, this may not always indicate that there are histological lesions in the muscle fiber membrane [31,32], and the consensus that it does not adapt to high load exercise is the basis for the impairment of the permeability of the cell membrane, thereby increasing serum levels of CK.

In addition to CK, elevated plasma LDH is an important biomarker of muscle damage induced by exhaustive exercise. Increases in serum LDH can also be a reflection of the production of ROS. The ROS are metabolites resulting from exercise, and one of the factors involved in reducing the contractile activity and causing muscle fatigue [33] when they are above the organic capacity for neutralization. In this sense, supplementation with extracts rich in antioxidant compounds can exert greater cellular resistance against oxidative stress [34], increase the activity of antioxidant enzymes, or stabilize the free radicals generated.

## 5. Conclusions

Regardless of whether it is a complex sample in which the predominant compounds are not fully identified, although chromatographic analysis has pointed to the presence of flavonoids, the HEE *C. argyrophyllus* showed an ability to partially reduce the biomarkers of oxidative stress at the plasma level and in the muscle. Taking into account CK and LDH activities, the HEE was able to induce reductions. These results provide a new perspective for the use of compounds derived from medicinal plants to prevent or combat muscle damage. For a better redox protection, it is necessary to use more purified fractions.

The design of this research was a limiting factor in the present study, as the evaluations were made only at the end of the intervention. Given that the tests are performed by the sacrifice of the animals, it does not allow for initial evaluation. Therefore, further research with initial data is important as a way to obtain a better experimental design for the study.

## Figures and Tables

**Table 1:** Antioxidant activity of the hydroethanolic extract of *C. argyrophyllus* determined by a DPPH test.

Samples	IP%	EC_50_ (µg/mL ± SEM)	AAI
HEE	39.79	243.8 ± 0.25 *	0.12
Gallicacid	87.61	8.2 ± 0.07 *	4.87

IP = inhibitory potential; AAI = antioxidant activity index, HEE = hydroethanolic extract. * *p* < 0.05.
